# Implementation of an older person’s nurse practitioner in rural aged care in Victoria, Australia: a qualitative study

**DOI:** 10.1186/s12960-019-0415-z

**Published:** 2019-11-01

**Authors:** Kaye Ervin, Carol Reid, Anna Moran, Cynthia Opie, Helen Haines

**Affiliations:** University of Melbourne, Melbourne, Australia

**Keywords:** Rural aged care, Nurse practitioner, Implementation

## Abstract

**Background:**

There are staff shortages nation-wide in residential aged care, which is only predicted to grow as the population ages in Australia. The aged care staff shortage is compounded in rural and remote areas where the health service workforce overall experiences difficulties in recruitment and retention. There is evidence that nurse practitioners fill important service gaps in aged care and rural health care but also evidence that barriers exist in introducing this extended practice role.

**Methods:**

In 2018, 58 medical and direct care staff participated in interviews and focus groups about the implementation of an older person’s nurse practitioner (OPNP) in aged care. All 58 interviewees had previously or currently worked in an aged care setting where the OPNP delivered services.

The interviews were analysed using May’s implementation theory framework to better understand staff perceptions of barriers and enablers when an OPNP was introduced to the workplace.

**Results:**

The major perceived barrier to capacity of implementing the OPNP was a lack of material resources, namely funding of the role given the OPNP’s limited ability to self-fund through access to the Medicare Benefits Schedule (MBS). Staff perceived that benefits included timely access to care for residents, hospital avoidance and improved resident health outcomes.

**Conclusion:**

Despite staff perceptions of more timely access to care for residents and improved outcomes, widespread implementation of the OPNP role may be hampered by a poor understanding of the role of an OPNP and the legislative requirement for a collaborative arrangement with a medical practitioner as well as limited access to the MBS.

This study was not a registered trial.

## Background

Similar to other countries, Australia is facing unprecedented challenges to meet the growing healthcare needs of an ageing population. Currently, just over 250 000 people utilise aged care services [1], but it is predicted that by 2050, over 3.5 million Australians will be accessing aged care services annually [2]. Despite this growth in demand, general medical practitioners in Australia report intention to decrease or stop providing services to people in residential aged care [3]. Workforce shortages in aged care settings are well known, in addition to the difficulty in recruiting the skill mix required to meet the increasingly complex needs of ageing people. Challenges in meeting workforce requirements for safe and quality care are amplified outside metropolitan areas, particularly in remote areas [4]. Nurse practitioners are well placed to fill this widening gap, but to be successful, these roles need to be promoted with managers and decision-makers who may have a limited understanding of the importance of the clinical support offered by the extended scope of practice [5].

Health workforce shortages across all disciplines in rural Australia have necessitated the implementation of a range of workforce models, including the use of nurse practitioners, as a strategy to improve access, efficiency and quality of care for patients [6]. Currently, there are around 1745 endorsed nurse practitioners in Australia, including 365 in the state of Victoria, working across a variety of specialty areas [7] including aged care.

Nurse practitioners are defined as registered nurses who possess expert knowledge, complex decision-making skills and clinical competence [8] with legislated expanded scope of practice to include diagnosis, prescribing and referral. In 2010, nurse practitioners were granted access to the public health insurance scheme (Medicare) subject to a collaborative arrangement with a medical practitioner [9] .

With access to Medicare, specialist aged care services are being increasingly provided by nurse practitioners [2] using various models of care, including independent private practice, outreach services from acute settings or working from community-based organisations. Nurse practitioners are providing services to all health settings, especially in rural and remote locations where there are workforce and skills shortages [5].

There is a paucity of evidence that is reported in the national and international literature regarding implementation of the nurse practitioner role, in particular in rural areas. Little is known about the challenges faced by owners or managers, or direct care staff, of rural residential aged care services in introducing a nurse practitioner role. The purpose of this study was to explore perceived barriers and enablers to implementing an older person’s nurse practitioner (OPNP) from the perception of residential aged care staff in rural aged care settings. The overall aim of the research is to describe the mechanisms which may be required for successful implementation of an OPNP role in rural residential aged care settings.

### Implementation framework

The theoretical framework chosen for the study was May’s implementation theory [10]. May’s theory considers the complex, multi-dimensional aspects of health services in relation to the introduction of a new intervention and provides a structure to better examine what does or does not work. Briefly, May’s implementation theory focuses on four main themes; capability, capacity, potential and contribution. These themes are defined as

1. Capability—the capability of agents to operationalise a complex intervention which depends on its workability and integration within a social system;
2. Capacity—the incorporation of a complex intervention within a social system which depends on an agent’s capacity to cooperate and coordinate their actions;
3. Potential—the translation of capacity into collective action which depends on an agent’s potential to enact the complex intervention; and
4. Contribution—the implementation of a complex intervention which depends on an agent’s continuous contributions that carry forward in time and space.

In the context of this study, agents are the staff of the residential aged care facility (RACF), including clinical care staff, general practitioners (GPs) and managers. The complex intervention is the OPNP role and scope of practice. The social system is the RACF.

## Methods

### Design

This study employed a qualitative research design using semi-structured interviews. The study scope was defined as sites where an OPNP delivered services, targeting health service staff associated with the implementation and delivery of the OPNP extended scope of practice. A purposive sample of health service management, care managers, nursing and care staff and GPs was sought. Qualitative data were generated through both focus groups and individual face to face or telephone interviews.

### Recruitment

Participant recruitment focussed on six [6] RACFs where the OPNP was contracted to deliver services. The participants were recruited through direct contact by both phone and email. Mutually suitable interview times and dates were then scheduled. All interviews occurred at participants’ workplaces in 2018. A brief description of the project was provided to gauge interest. Those who expressed interest in participating were provided with a plain language statement and a consent form to sign.

### Intervention

Each of the RACFs in the study contracted the OPNP privately, with varying contracted hours (shown in Table 1). In addition, the period of time that the OPNP had been delivering services differed between sites as also shown in Table 1. As well as a collaborative agreement with GPs, at each site the OPNP is mentored by a geriatrician. The OPNP scope of practice includes access to diagnostics (such as radiology and pathology), prescribing (with limited access to the Pharmaceutical Benefits Scheme (PBS) depending on approval) and referral to specialist medical professionals. Access to the Medicare Benefits Schedule (MSBS)^1^ for payment is limited and varies according to clinical practice endorsement. The OPNP provided the research team with an estimate of the percentage of older people receiving service at each site (Table 1). Service delivery percentages vary over time according to admission and discharge (death) of older people in the facility and their treating GP.

**Table 1:** Recruitment sites

Sites	RACF service type	Bed numbers available	Services available in township (town population)	Privately contracted hours of OPNP	Approximate percentage of residents with GP collaborative agreement	Period of time of service delivery
Site 1	Public sector—2 facilities	36 15	Acute service Urgent Care Allied health Community services Medical clinic (population = 1 082)	8 h per week	100%	1.5 years
Site 2	Not for profit	146	Regional centre with all medical and allied health services (population = 28 559)	8 h per week—divided into two 4-h sessions	50%	6 years
Site 3	Not for profit	69	Regional centre with all medical and allied health services (population = 28 559)	12 h per week—divided into three 4-h sessions	60%	6 years
Site 4	Public sector	14	Acute service Urgent Care Community services Medical clinic (population = 406)	8 h per month	100%	2.5 years
Site 5	Public sector—2 facilities	30 32	Acute service Operating theatre Radiology Pathology Urgent care Community services Medical clinic × 2 (population = 4 477)	8 h per fortnight—divided into 4 h per week	100%	1 year

### Settings

This study involved five geographical sites (one regional and four rural), in Victoria, Australia. A description of the de-identified recruitment sites are shown in Table 1 with an accompanying description of the services available in the location and overall population of the location. Public sector RACFs are wholly funded by the government, and not for profit RACFs receive partial funding and as the name suggests are provided by charitable organisations not private organisations that aim to generate a profit.

### Interviews

The interviews and focus groups were conducted by two female researchers in 2018. One researcher is a nurse (CO) and one a social worker (CR). Both have qualitative research experience. Some interview participants at one rural site were known to one researcher (CR). A copy of the interview schedule is available from the corresponding author. In brief, participants were asked two key questions:

- What is the experience of implementing an OPNP?
- What are the enablers and barriers to implementing an OPNP in RACFs?

Sub-questions included understanding of the NP role and management motivation for implementation.

### Data analysis

Participant interviews were initially grouped by site, because of the dynamic element of context in implementation theory. A number of the GP participants worked across more than one site; therefore, interviews were re-grouped and GPs assigned as a separate group. Final analysis was undertaken on six groups. The final de-identified groupings and their composition and total number are shown in Table 2.

**Table 2:** Final analysis groups and composition

Site or group	Description	Total participant number by site
Site 1	Health service focus group Director of clinical services Quality and risk manager Medical clinic staff focus group	10
Site 2	RACF focus group RACF clinical care coordinators × 2 Registered nurse RACF Team leader RACF	8
Site 3	Registered nurse RACF × 2 Enrolled nurse × 2 RACF manager RACF focus group	10
GP group	GPs × 4 GP focus group sites 2 and 3	7
Site 4	Registered nurse health service × 2 Director of clinical services Registered nurse medical clinic	4
Site 5	RACF focus group × 2 RACF clinical care coordinators × 2 Director of RACF RACF manager Health service manager Health service director of clinical services	19

All transcripts were analysed by hand. Preliminary, first cycle coding used process and values coding as an elemental method to attune researchers to participants’ perspectives and actions [11]. This was undertaken by three researchers (CR, HH, AM). Second cycle coding (theoretical coding) was undertaken by two researchers (KE and CR) utilising May’s implementation theory framework [10] (see Table 3 in “Results” for a concise description of this theory). Codes from implementation theory domains were generated and sub-categories applied to tracts in text that reflected these concepts. This enabled the whole dataset to be indexed into domains and descriptive sub-categories. Content analysis [12] of positive and negative perceptions allowed identification of enablers or barriers to implementation.

**Table 3:** Implementation theory domains and sub-categories [10] with quotations

Domains	Subcategories	Perceived barriers (exemplifying negative quotations)	Perceived enablers (exemplifying positive quotations)
*Capability* The capability of agents to operationalise a complex intervention depends on its workability and integration within a social system.	*Workability*: the social practices that agents perform when they operationalise a complex intervention within a social system, and characterises interactions between users and components of a complex intervention. *Integration*: the linkages that agents make between the social practices of a complex intervention and elements of the social system in which it is located, and characterises interactions between the context of use and components of a complex intervention.	“I mean obviously to try and get the GPs to work collaboratively was probably the biggest hurdle and it is still the biggest hurdle” [staff member] “We are not aware of [OPNP] scope of practice in the first place, that’s our trouble” [focus group participant] “… .it wasn’t implemented well and that may be why we had a lot of issues at the start, because we did not really understand it …” [staff member] “It was hard to know what your position was … an understanding of what your roles will be” [staff member] “Whether the particular need [for an OPNP] was identified I have no idea …” [staff member]	“… given that we are a smaller health service here and the GPs are on site, we have that ability to actually get that partnership and that collaboration working really well” [staff member]
*Capacity* The incorporation of a complex intervention within a social system depends on agents’ capacity to cooperate and coordinate their actions.	*Social norms*: institutionally sanctioned rules that give structure to meanings and relations within a social system, and that govern agents’ membership, behaviour and rewards within it. They frame rules of membership and participation in a complex intervention. *Social roles*: socially patterned identities that are assumed by agents within a social system, and that frame interactions and modes of behaviour. They define expectations of participants in a complex intervention. *Material resources*: symbolic and actual currencies, artefacts, physical systems, environments that reside within in a social system and that are institutionally sanctioned, distributed and allocated to agents. They frame participants’ access to those material resources needed to operationalise the complex intervention. *Cognitive resources*: personal and interpersonal sensations and knowledge, information and evidence, real and virtual objects that reside in a social system and that are institutionally sanctioned, distributed and allocated to agents. They frame participants’ access to knowledge and information needed to operationalise the complex intervention.	“… I do not know whether it’s [OPNP role] accepted, or does not have its rightful place in the structure of health care” [staff member] “I think it deskills some of our nursing staff because they seem to think they cannot do anything now without getting a nurse practitioner involved” [GP] “… there is still times when one of the GPs in particular feels that potentially the nurse practitioners might be overstepping the mark” [focus group member] “… they are [GPs] trying to micromanage [OPNP] and I think that’s not allowing [OPNP] to practice to [OPNP] full capacity” [staff member] “The funding models are not there” [staff member] “So funding in certain places has been difficult to get. One aged care facility said ‘well we cannot afford this anymore’” [GP group member] “Having to find additional funds to support [OPNP] is a big issue … it’s going to be very much influenced by what we can afford to do …” [staff member] “… the residents already have practitioners who are quite involved in their care so I think that perhaps for this area it [OPNP] might be a trifle superfluous” [GP group member]	“As the GPs have understood – learnt and understood more about nurse practitioner roles they have become more comfortable with letting go” [focus group member] “[OPNP] is also a good resource person …. [OPNP] is a very good link to all those other services” [focus group member] “… employing a nurse practitioner here was predominantly to fill [delay in treatment] gap … prevent them going to hospital unnecessarily …” [staff member] “That makes it quite – I mean, or job, quite a bit easier” [GP group member] “… having a nurse practitioner brings an additional level of knowledge into the organisation …” [staff member]
*Potential* The translation of capacity into collective action depends on agents’ potential to enact the complex intervention.	*Individual intentions*: agents’ readiness to translate individual beliefs and attitudes into behaviours that are congruent, or not congruent, with system norms and roles. They frame individual motivation to participate in a complex intervention. *Shared commitments*: agents’ readiness to translate shared beliefs and attitudes into behaviours that are congruent, or not congruent, with system norms and roles. They frame shared commitment of participation in a complex intervention.	“I have no reason to dislike [OPNP] … but I do not feel any connection to go and ask for help or seek [OPNP] opinion” [staff member] “It has not been embraced from either side probably as well as what it could have been” [staff member]	“The good communication between bodies, staff and [OPNP] or [OPNP] is part of the staff anyway, so it’s reciprocated, that respect” [focus group member] “Everything [OPNP] does he involves people” [staff member]
*Contribution* The implementation of a complex intervention depends on agents’ continuous contributions that carry forward in time and space.	*Coherence or sense-making*: agents attribute meaning to a complex intervention and make sense of its possibilities within their field of agency. They frame how participants make sense of, and specify their involvement in a complex intervention. *Cognitive participation*: agents legitimise and enrol themselves and others into a complex intervention. They frame how participants become members of a specific community of practice. *Collective action*: agents mobilise skills and resources and enact a complex intervention. They frame how participants realise and perform the intervention in practice. *Reflexive monitoring*: agents assemble and appraise information about the effects of a complex intervention within their field of agency, and utilise that knowledge to reconfigure social relations and action. They frame how participants collect and utilise information about the effects of the intervention.		“Families are certainly very, very keen on it [OPNP]” [staff member] “… we were auditing … I could see the team involvement in the care of residents, especially in their end state of care with the doctors and the GP and the nurse practitioner being involved” [staff member] “You can actually see the results in the clinical indicators … huge improvement … because you are getting better intervention much earlier” [focus group member] “We are reducing [accident and emergency presentations] here because we have [OPNP] on site” [staff member]

#### Implementation theory framework

It is not enough to know if a health intervention is effective; it is also necessary to understand why an intervention works, how, for whom and in what context. Implementation science is aimed at mobilising theories, concepts and methods to better understand what, why and how interventions work in the “real world” [13]. To be more precise in the description of the analysis of our qualitative study, it was important to better describe the content and ongoing processes of implementation of the OPNP role for fidelity of the findings. May’s implementation theory provided a framework to index the domains and sub-categories for a more thorough understanding of what worked and how it worked. Implementation theory and frameworks are increasingly being used in contextual analysis and research designs in order to have a greater understanding of how to implement health interventions and policy. The World Health Organization encourages the use of implementation science to create better adaption and adoption of health interventions [14].

## Results

Fourteen hours and fifteen minutes of voice recorded material was transcribed verbatim. Transcripts were returned to participants who consented to check for authenticity.

The findings presented are from across all sites, and categorised as barriers and enablers to implementation of an OPNP in residential aged care. The findings are presented under the domains of implementation theory: capability, capacity, potential and contribution. Each of these domains is then described by sub-categories and participant quotes to better illustrate how barriers and enablers were derived from positive and negative perceptions.

There were equivalent amounts (*n* = 77) of positive and negative perceptions illustrating barriers and enablers regarding capability as a measure of implementation. At all sites, the legislative requirement of an OPNP working in a collaborative arrangement with a GP was perceived as a barrier, except at the smallest health service, where it was considered to work well. A lack of information and partnership with staff regarding the introduction of the OPNP role and scope of practice was perceived negatively and translated as a barrier for implementation of the role. Field notes illustrate that staff at some sites felt undermined by the introduction of the OPNP, that their skills were insufficient or that some “need” had been identified but had not been communicated to them. Integration into practice also embodies assumptions about an intervention and its expected value.

Positive (*n* = 262) and negative (*n* = 267) perceptions of capacity to implement the OPNP role were identified in the analysis. Social roles and material resources were the most dominant feature perceived as barriers and enablers. Material resources were perceived as contingent as an enabler to employ an OPNP. Social roles, the interpersonal relationship between the OPNP and GPs and the OPNP and RNs, were frequently perceived negatively and therefore identified in the analysis as a barrier to implementation of the OPNP role, though this was also identified as something that ameliorated over time.

The potential to implement the OPNP role when analysed using implementation theory was predominantly negative (*n* = 28) when compared to positive perceptions (*n* = 12) and therefore collectively identified as a barrier. The two quotations exemplifying enablers relate to subcategory of shared commitment.

Reflexive monitoring in implementation of the OPNP role was identified as an enabler in terms of contribution, with multiple positive perceptions of the OPNP role when auditing outcomes of resident care with three quotes illustrating this. Overall, there were 29 positive comments related to contribution.

## Discussion

### Capability

Workability as a concept of implementation of an older person’s nurse practitioner (OPNP) was perceived as problematic at almost all the research sites involved in this study. In particular, the legislated requirement for a collaborative arrangement between nurse practitioners and medical practitioners was viewed negatively. A previous Australian study [9] found that success of the collaborative arrangement relied on the personal commitment and willingness of both NPs and GPs. Schadewaldt et al. [9] also found more complex problems with the legislative requirements, such as disadvantages for NPs if the local infrastructure did not include a visiting GP or overlapping of roles and blurring of professional boundaries and legal liability. Collaboration is also influenced by interpersonal differences of NPs and GPs, which was found in this study and reported in a systematic review of NP experiences [15]. There have been allegations that the poor uptake of NP training and implementation of the role can be ascribed to medical dominance and a power struggle between nurses and GPs about professional roles [16]. It is a legislative prerequisite for NPs to have a collaborative agreement with a medical practitioner in order to access Medicare subsidy schemes. The Medicare Benefits Schedule Review Taskforce [17] is currently reviewing the requirement for NPs to have a collaborative agreement with a medical practitioner. Revision of this legislation could remove a significant barrier to implementation of OPNP roles found in this study.

Barriers to integration of the OPNP role was reported at many of the study sites and focused predominantly on staff reporting a poor knowledge of the NP role and scope of practice. Other studies [17, 18] also cite the paucity of healthcare professionals understanding of the NP role, which in turn impacted on NPs being able to work at their full scope of practice. Dwyer et al. [19] report that knowledge of and prior exposure to the NP role was a contributing factor to successful implementation of the role, which is also supported by the findings of a more recent study [5]. May [10] warns that successful implementation of any intervention is threatened if the capacity of staff to employ it is confounded. To put it simply, successful implementation of the OPNP requires aged care staff to have a good understanding of the extended scope of practice. One study [20] reported that holding meetings with staff to clarify questions and dispel concerns about NPs was key to successful implementation. The reported paucity of staff knowledge of the NP role suggests that NPs themselves have a responsibility to ensure co-workers have a good understanding of their scope of practice and the limitations of their role. It can be assumed that this is even more crucial when OPNPs are employed at RACFs where staff have had no previous exposure to the NP role. Staff must feel engaged during the implementation process to ensure an understanding that the new position is “adding value” rather than “replacing” existing roles.

### Capacity

Staff of the RACFs participating in this study reported positive perceptions about the benefits for staff and residents afforded by the availability of an OPNP, while being largely negative about the financial ability to employ them and the blurring of professional boundaries.

Dwyer et al. [19] cite multiple studies that demonstrate holistic NP-led models of care that engender a positive impact upon residents’ quality of life and health outcomes, in addition to reducing hospital admissions, which was also found in this study. In this study, staff attributed this outcome to the availability of the OPNP to treat residents, rather than waiting extended periods of time for a GP to be available. This is supported by the findings of Dwyer et al. that early intervention by NPs led to timely treatment and subsequent hospital avoidance which met the needs of the resident, family and RACF staff.

Currently, NPs in Australia have limited access to the MBS subsidy schemes. While medical practitioners have access to remuneration for hundreds of MBS items, NPs are limited to four items and reimbursement is at 85% of the scheduled fee. These fiscal constraints mean that NPs either have to charge residents directly or be employed by an organisation for service delivery. Even access to the four items on the MBS is dependent upon a collaborative arrangement with a medical practitioner. NPs are therefore reliant on organisations funding their positions within the organisations’ own budgetary constraints. The participants in this study who were responsible for employing the OPNP stated that they did so because of prior exposure to the role and scope of the NP and perceived that the associated costs of the NP wages translated into improved quality of care for the services delivered. The implication of this is that the capacity of a RACF to employ an OPNP is reliant on material resources for both its introduction and sustainability. May [10] proposes that capability, particularly normalisation into practice, is threatened through poor resourcing or uncertain sustainability. A review of international studies found that one reason for underutilisation of NPs included a lack of financial support [21]. The most recent Australian study [5] found that even when funding for a NP role was available, it was not recurrent, in which case the positions went unfilled. Another Australian study found that the difficulties faced by NPs in generating their own income decreased their chances of employment [20]. The Medicare Benefits Schedule Review Taskforce [17] may remove this financial barrier to implementation; if access to an increased number of MBS items occurred, NPs could be better remunerated for their services, negating the need to be employed by RACFs as private providers.

The blurring of professional boundaries in the NP scope of practice is a very real concern, particularly regarding legal liability. Some participants in this study expressed concern and uncertainty about the implications for their scope of practice once the OPNP was introduced. It was perceived that their scope would be reduced, or to continue to perform tasks in their usual role would “step on toes”. Other participants also expressed that the introduction of the OPNP resulted in deskilling of the usual staff, who became reluctant to work to their full scope of practice due to uncertainty about what was now acceptable. This could be resolved through better education of existing staff [20] or adhering to important principles of workforce change [22] prior to implementation of the OPNP role.

Some GP participants in this study perceived that they were ultimately legally responsible for the care of residents. This may be perpetuated by the legislated collaborative requirement. Although professional guidelines in Australia clearly state that individual practitioners are responsible for their own actions [23, 24], contrasting perceptions are demonstrated in practice. This study supports the finding of Schadewaldt et al.’s study [20] that medico-legal liability is unclear when patient care is shared between NPs and medical practitioners. There needs to be clear legislation to resolve this discrepancy with shared care and for NPs to be truly independent and autonomous in their delivery of care and treatment in RACFs.

### Potential

In this study, the sub-categories of potential, namely collective and individual commitment, were largely perceived negatively. This may stem from poor understanding of the OPNP role and scope of practice. It is difficult to support a role and model of care when there is little conceptual understanding of the purpose of the role and the processes and limitations of utilising the role. It is difficult to form a discussion around this generally given the many different models of NP services. In this study, and for OPNPs who provide services on a contractual basis to RACFs, potential could be facilitated by prioritising information about the NP role through staff education and communicating the clear aim of introducing the role. Dwyer et al. [19] propose that potential develops over time with increased exposure and building trust between stakeholders and the OPNP. It is also proposed that building trust, particularly in rural areas, is not simply acceptance of formal qualifications but more about an unwritten need to “prove yourself” [19]. Trust was exemplified in this study, where participants at sites 2 and 3 (where the OPNP had been practising for longer) expressed far more positive perceptions about the role and the individual OPNP, as well as an intention to maintain the OPNP service. The implication of this finding is that caution should be exercised by RACFs to ensure the individual NP is a good “fit” with the organisation.

Conversely, Dwyer et al. [19] also found that NPs have the unique potential to facilitate collective and individual commitment by their knowledge of dual professions, systems and close professional relationship with residents, which enabled them to become a conduit between groups. Findings from this study also supports the findings of Dwyer et al.’s study [19] that RACF staff were more confident discussing resident care with a NP than a GP, because they were nurses and therefore “speak the same language”. However, this finding was only true in this study at two sites where the OPNP had practised for a greater length of time, suggesting this was also related to time or trust.

### Contribution

At the study sites where reflexive monitoring had occurred through auditing of impact of the role compared with resident outcomes, perceptions of contribution were positive. Comments related to contribution were not elicited at one site; interestingly, that site had been utilising the OPNP for the least amount of time, potentially indicating that the length of service provision had not yet allowed reflexive monitoring. At all other sites where reflexive monitoring had occurred, comments reflected perceived benefits. Like other studies [25, 26], this study found that staff perceived the implementation of the OPNP role had a direct effect on improved quality of care. In this study, staff perceptions were based on the results of auditing quality outcomes such as reduced hospital transfers. This finding implies that implementation of an OPNP in RACFs may translate into cost-effective savings for the entire health system.

Other studies [18, 27] found that a poor understanding of the NP role influenced consumers’ decision to see a NP. In this study, there was demand from residents to see the NP due to awareness of the role. Demand was not able to be met because the resident’s treating GP did not have a collaborative agreement with the NP. Demand for NP services has not been previously reported in the literature, so consumer demand for an OPNP is a unique finding of this study. This finding has important implications for aged care policy makers, with a focus on increasing choices for older people ubiquitous in policy, legislation and standards of care. Demand for an OPNP should also be considered in light of the decreasing access to medical services in RACFs and the intention of Australian GPs to decrease or cease services to RACFs [3].

The benefit of using May’s implementation theory [10] for this study was an interpretation of agent perceptions across six RACF sites regarding the introduction of an OPNP model of care. Using implementation theory as a framework when considering new models of care in any context is crucial in all stages of planning from needs assessment, theory of change development to theory of action (implementation).

A limitation of this study was that interviews were conducted only with staff of RACFs and did not include perceptions of residents or their families. This limitation may have missed an opportunity to further explore consumer demand for OPNP services. Additionally, the study was conducted in rural and regional RACFs and the findings may not be transferable to metropolitan settings.

## Conclusion

Implementation of any health care intervention is complex, and success is reliant on multiple factors. This study was undertaken to explore all the complex factors related to implementing an OPNP in RACFs in rural Victoria in order for RACF managers to understand the resources and processes that may be required for successful implementation and for policy makers to understand the perceived barriers and thereby address them. The Medicare Benefits Schedule Review Taskforce may provide solutions to the major barriers if the recommendations for greater access to the MBS for NPs, and review of the collaborative arrangement between NPs and medical practitioners are fully considered. Both recommendations would resolve perceived barriers found in this study, predominantly remuneration for NP services and reliance on a consenting GP for access to and professional collaboration for treatment of people in RACFs.

## Data Availability

A copy of the interview schedule and de-identified transcripts are available from the corresponding author. Due to the context of the study in small rural RACFs, original transcripts of interviews are withheld to protect participants’ identities.

## Abbreviations

GP: General practitioner
MBS: Medicare Benefits Schedule
NPs: Nurse practitioners
OPNP: Older person’s nurse practitioner
RACF: Residential aged care facility
RN: Registered nurse

## Footnotes

1 Medicare is Australia’s government health care system. Employed people pay a Medicare levy as part of their income tax to fund the system for all Australians requiring medical treatment. Government-funded medical services are listed on the MBS. The government authorises which practitioners can claim the government funding for providing services. Most services provided by a medical practitioner are funded wholly or partly by Medicare. Nurses and allied health practitioners have restricted access to the MBS for providing services.
